# Association Between Breastfeeding and Dental Caries in Japanese Children

**DOI:** 10.2188/jea.JE20110042

**Published:** 2012-01-05

**Authors:** Keiko Tanaka, Yoshihiro Miyake

**Affiliations:** Department of Preventive Medicine and Public Health, Faculty of Medicine, Fukuoka University, Fukuoka, Japan

**Keywords:** breastfeeding, cross-sectional studies, dental caries, Japan

## Abstract

**Background:**

Studies investigating the impact of breastfeeding on dental caries have produced contradictory results. This cross-sectional study investigated the relationship between breastfeeding and the prevalence of dental caries in young Japanese children.

**Methods:**

The study subjects were 2056 Japanese children aged 3 years. Information on breastfeeding was obtained by means of a questionnaire. Children were classified as having caries if 1 or more deciduous teeth were decayed, missing, or had been filled at the time of examination.

**Results:**

The prevalence of dental caries was 20.7%. As compared with breastfeeding for less than 6 months, breastfeeding for 18 months or longer was associated with a significantly higher prevalence of dental caries. The relation was J-shaped: the adjusted prevalence ratios for less than 6 months, 6 to 11 months, 12 to 17 months, and 18 months or longer were 1.0, 0.79 (95% confidence interval [CI]: 0.60–1.05), 0.86 (95% CI: 0.66–1.13), and 1.66 (95% CI: 1.33–2.06), respectively (*P* for linear trend <0.0001, *P* for quadratic trend <0.0001).

**Conclusions:**

Breastfeeding for 18 months or longer was positively associated with the prevalence of dental caries, while breastfeeding for 6 to 17 months was nonsignificantly inversely associated with the prevalence of dental caries.

## INTRODUCTION

Breastfeeding is promoted as the preferred method of infant feeding and provides a number of advantages, including health, nutritional, immunological, developmental, psychological, social, economic, and environmental benefits.^1^

However, the evidence has been mixed regarding the association between breastfeeding and dental caries.^2^^–^^18^ Several studies have reported an inverse association between breastfeeding and dental caries,^2^^–^^4^ while other studies have failed to show any highly beneficial association.^5^^–^^13^ Moreover, some studies have demonstrated that breastfeeding was associated with a higher prevalence of dental caries.^2^^,^^10^^,^^13^^–^^18^ A US cross-sectional study in children aged 2 to 5 years showed that breastfeeding and its duration were not associated with an increased prevalence of early childhood caries.^5^ In a retrospective cohort study of early childhood caries among infants aged 25 to 30 months in Myanmar, the prevalence of early childhood caries was greater in children who were breastfed more than twice at night, whereas there was no association between daytime breastfeeding habits and prevalence.^10^

These inconsistent and even contradictory results among studies are likely mostly due to methodological differences, such as the use of different cut-off points for breastfeeding, lack of adjustment for confounding factors, different definitions of dental caries, and the ages at which outcomes were assessed. Additional data are required to reach a conclusion concerning the association between breastfeeding and dental caries. This cross-sectional study investigated the association between breastfeeding and the prevalence of dental caries among young Japanese children.

## METHODS

### Study population

Data for the present study came from the Fukuoka Child Health Study (FCHS), a cross-sectional study of the association between various selected factors and child health problems, such as dental caries and allergic disorders.^19^^–^^22^ In Japan, when children reach age 3 years, the municipality in which the family currently resides sponsors a physical examination that includes a dental examination, measurement of height and weight, and an interview with parents or guardians regarding the child’s health. Eligible subjects for the present study were children aged 3 years who received this physical examination at any of the 7 public health centers offering it in Fukuoka City, a metropolitan area on Kyushu Island in southern Japan with a total population of approximately 1 414 000. During the period from June 2006 to January 2007, we were granted permission by the Fukuoka City government to provide our questionnaires directly to parents or guardians of children receiving the physical examination at age 3 years. Out of the 8269 eligible children, the parents or guardians of 8064 were provided with a structured self-administered questionnaire, a brief self-administered diet history questionnaire (4 pages), and a postage-paid, addressed, return envelope. The structured self-administered questionnaire consisted of 16 pages: 13 pages for the 68 questions and 3 pages for data transcription from the Maternal and Child Health Handbook. Ultimately, the parents or guardians of 2109 children answered the questionnaires and mailed these materials to the data management center (participation rate = 25.5%). Our research technicians conducted telephone interviews with individual participants when it was necessary to obtain missing data or clarify implausible responses. The current study was restricted to subjects who provided complete information concerning the variables under study; this left 2056 children available for analysis (24.9% of all eligible children). Permission to perform this study was obtained from the ethics committee of the Faculty of Medicine at Fukuoka University.

### Outcome variable

During the physical examination, the presence of dental caries was assessed by visual examination without the use of radiographs. Dental examination data were recorded by a dentist in the Maternal and Child Health Handbook provided by the municipality during pregnancy, in which data pertaining to prenatal checkups, postnatal health conditions of both mother and baby, and growth of the child are recorded.^23^ The first half of the handbook provides space for recording data on health condition and vaccination records, and the latter half provides information for mothers on pregnancy, delivery, and parenting. In our study, the parents or guardians of the children had to transcribe dental examination data from the Maternal and Child Health Handbook to our self-administered questionnaire. To facilitate the transcription of the oral examination data to the self-administered questionnaire, we used exactly the same format as that used for the records in the Maternal and Child Health Handbook, and parents or guardians transcribed all of the information, including symbols. Children were classified as having dental caries if 1 or more primary teeth had decayed, were missing, or had been filled.

In a sensitivity analysis, we also used data on dental caries gathered at 18 months of age, which were recorded by a dentist in the Maternal and Child Health Handbook when our subjects received their municipal physical examinations at age 18 months. These data were transcribed by parents or guardians from the Maternal and Child Health Handbook to our questionnaire.

### Exposure variables and covariates

Information on breastfeeding duration in months was obtained from the structured self-administered questionnaire described above. Breastfeeding duration was the period during which the infants received breast milk, regardless of exclusivity. The questionnaire also included questions on the sex of the child, dental health practice (such as toothbrushing frequency, use of fluoride, and pattern of dental care), between-meal snack habits, maternal smoking during pregnancy, environmental tobacco smoke (ETS) exposure at home, and paternal and maternal educational levels. Use of fluoride was defined as positive if children reported using fluoride toothpaste or receiving topical application of fluoride gel at a health center or a dental clinic.

Data from the brief self-administered diet history questionnaire were not used in the current study.

### Statistical analyses

Breastfeeding duration was classified into 4 categories (<6 months, 6–11 months, 12–17 months, and ≥18 months). The following variables were controlled for in the multivariable model: the sex of the child, toothbrushing frequency (<2 or ≥2 times/day), use of fluoride (yes or no), regular dental check-ups (yes or no), between-meal snack frequency (<1, 1, or ≥2 times/day), maternal smoking during pregnancy (yes or no), ETS exposure at home (yes or no), and paternal and maternal educational level (<13 years, 13–14 years, and ≥15 years). In the sensitivity analysis using data on dental caries at age 18 months, adjustment was made for child sex and parental educational levels only. Prevalence ratios (PRs) and 95% confidence intervals (CIs) were estimated using binomial regression with the log link function.^24^ Linear trend of the association between duration of breastfeeding and dental caries was assessed using a log-binomial regression model, treating the categories of breastfeeding duration as consecutive integers. For tests of quadratic trend, we included linear and quadratic terms in the model. Two-sided *P* values less than 0.05 were considered to indicate statistical significance. All statistical analyses were performed using the SAS software package version 9.1 (SAS Institute, Cary, NC, USA).

## RESULTS

Of the 2056 children, 425 (20.7%) had dental caries. The mean number of dental caries was 0.70. Toothbrushing 2 or more times per day was reported in about 40% of the subjects. Approximately 85% of children reported fluoride use, either as fluoride toothpaste or topical application of fluoride gel at a health center or a dental clinic. Approximately 44% of children received regular dental check-ups. More than 41% of subjects had between-meal snacks 2 or more times per day. In utero exposure to maternal smoking occurred in 13.1% of children, and 43.8% were exposed to ETS at home at least once. About 20% of children were breastfed for less than 6 months, whereas 27.2% were breastfed for 18 months or longer.

Table 1 shows the results of bivariate analyses for selected covariates. Regular dental check-ups were associated with a lower prevalence of dental caries. In contrast, 2 or more between-meal snacks per day was positively associated with dental caries. With regard to smoking, both maternal smoking during pregnancy and postnatal ETS exposure were associated with an increased prevalence of dental caries. Children with fathers who had more than 15 years of education and those with mothers who had 13 to 14 years, or more than 15 years, of education were less likely to have caries than children with fathers or mothers who had less than 13 years of education.

**Table 1.** Prevalence ratios (PRs) and 95% confidence intervals for dental caries in relation to study variables (unadjusted analysis)

	Prevalence	Crude PR (95% CI)
Sex		
Female	184/969 (19.0%)	1.00
Male	241/1087 (22.2%)	1.17 (0.98–1.39)
Toothbrushing frequency (times/day)		
<2	252/1244 (20.3%)	1.00
≥2	173/812 (21.3%)	1.05 (0.89–1.25)
Use of fluoride		
No	54/318 (17.0%)	1.00
Yes	371/1738 (21.4%)	1.26 (0.97–1.63)
Regular dental check-ups		
No	301/1166 (25.8%)	1.00
Yes	124/890 (13.9%)	0.54 (0.45–0.65)
Between-meal snack frequency (times/day)		
<1	78/444 (17.6%)	1.00
1	128/754 (17.0%)	0.97 (0.75–1.25)
≥2	219/858 (25.5%)	1.45 (1.15–1.83)
Maternal smoking during pregnancy		
No	346/1787 (19.4%)	1.00
Yes	79/269 (29.4%)	1.52 (1.23–1.87)
ETS exposure at home		
No	205/1156 (17.7%)	1.00
Yes	220/900 (24.4%)	1.38 (1.16–1.63)
Paternal educational level (years)		
<13	145/565 (25.7%)	1.00
13–14	63/307 (20.5%)	0.80 (0.62–1.04)
≥15	217/1184 (18.3%)	0.71 (0.59–0.86)
Maternal educational level (years)		
<13	166/581 (28.6%)	1.00
13–14	151/823 (18.4%)	0.64 (0.53–0.78)
≥15	108/652 (16.6%)	0.58 (0.47–0.72)

ETS, environmental tobacco smoke.

Table 2 shows the PRs and their 95% CIs for the relationship between breastfeeding duration and the prevalence of dental caries. As compared with less than 6 months of breastfeeding, breastfeeding for 18 months or longer was significantly associated with a higher prevalence of dental caries. After adjustment for sex, toothbrushing frequency, use of fluoride, dental check-up history, between-meal snack frequency, maternal smoking during pregnancy, ETS exposure at home, and paternal and maternal educational levels, the positive association remained statistically significant (adjusted PR = 1.66, 95% CI: 1.33–2.06). A J-shaped relationship was observed between breastfeeding duration and dental caries: the lowest PR was among children breastfed for 6 to 11 months (adjusted PR = 0.79, 95% CI: 0.60–1.05) and the highest PR was for children breastfed for 18 months or longer (adjusted PR = 1.66, 95% CI: 1.33–2.06, *P* for linear trend <0.0001, *P* for quadratic trend <0.0001).

**Table 2.** Prevalence ratios (PRs) and 95% confidence intervals for dental caries according to duration of breastfeeding in 2056 children (FCHS, Japan)

	Prevalence	Crude PR (95% CI)	Adjusted PR (95% CI)^a^
Breastfeeding duration (months)			
<6	85/416 (20.4%)	1.00	1.00
6–11	74/498 (14.9%)	0.73 (0.55–0.97)	0.79 (0.60–1.05)
12–17	90/583 (15.4%)	0.76 (0.58–0.99)	0.86 (0.66–1.13)
≥18	176/559 (31.5%)	1.54 (1.23–1.93)	1.66 (1.33–2.06)
*P* for linear trend		<0.0001	<0.0001
*P* for quadratic trend		<0.0001	<0.0001

FCHS, Fukuoka Child Health Study.

^a^Adjusted for sex, toothbrushing frequency, use of fluoride, regular dental check-ups, between-meal snack frequency, maternal smoking during pregnancy, exposure to environmental tobacco smoke at home, and paternal and maternal educational levels.

We also conducted a sensitivity analysis using dental caries at 18 months of age as the outcome variable. The prevalence of dental caries at age 18 months was 2.9% (*n* = 59). After adjustment for sex and paternal and maternal educational levels, breastfeeding duration was significantly and positively associated with dental caries in children at age 18 months. As compared with children breastfed for less than 6 months, the adjusted PRs for children breastfed for 6 to 11, 12 to 17, and 18 months or longer were 1.52 (95% CI: 0.45–5.17), 2.57 (95% CI: 0.85–7.82), and 6.45 (95% CI: 2.30–18.11), respectively (*P* for linear trend <0.0001).

## DISCUSSION

The present study found that breastfeeding for 18 months or longer was significantly associated with a higher prevalence of dental caries. Our results partially agree with those of other studies showing an adverse effect of breastfeeding on dental caries^2^^,^^10^^,^^13^^–^^18^ but are at variance with previous findings indicating a null or inverse association between breastfeeding and dental caries.^2^^–^^13^ We observed a J-shaped relationship between duration of breastfeeding and the prevalence of dental caries, ie, a nonsignificant inverse association was found between breastfeeding for 6 to 17 months and the prevalence of dental caries. Nevertheless, in a sensitivity analysis using data on dental caries at age 18 months, a nonsignificant positive association was observed between breastfeeding for 6 to 17 months and the prevalence of dental caries, while breastfeeding for 18 months or longer was significantly positively associated with the prevalence of dental caries. With regard to dental caries that had developed before age 18 months, the potential beneficial effects of breastfeeding for 6 to 17 months might not be detected. Alternatively, the results of the sensitivity analysis might have arisen by chance.

A systematic review suggested that breastfeeding for longer than 1 year, as well as nighttime breastfeeding after the eruption of teeth, is associated with some forms of early childhood caries, although the lack of methodological consistency and the inconsistent definitions of caries and breastfeeding used in previous studies make it difficult to draw definitive conclusions.^25^ For example, some studies compared breastfeeding for 13 months or longer with a period shorter than 13 months.^7^^,^^16^ Some studies investigated only the association between breastfeeding at night and dental caries.^6^^,^^10^^,^^18^ One study compared outcomes according to whether breastfeeding was ever performed or never performed.^12^ Inconsistency in the definition of dental caries, such as whether it comprises filled or missing teeth^16^; decayed, missing, or filled teeth^4^^–^^6^^,^^8^^,^^17^; 2 or more decayed, missing, or filled labial or palatal surfaces of primary incisors^11^; or cavitated, filled, or missing smooth surfaces in primary maxillary anterior teeth,^18^ also reduces the comparability of the available studies. Moreover, many studies were unable to control for important confounding factors such as oral hygiene practices,^4^^,^^6^^–^^8^^,^^10^^,^^11^^,^^16^^,^^18^ exposure to fluoride,^4^^–^^8^^,^^10^^,^^11^^,^^14^^,^^16^^,^^18^ and socioeconomic status.^4^^,^^8^^,^^10^^–^^12^^,^^16^^,^^18^

We do not have a definitive explanation regarding the mechanisms that underlie our observations. Several minerals in breast milk, such as phosphate and calcium, help protect tooth enamel. The mineral composition of breast milk changes with advancing lactation, which may affect its cariogenic properties. In a longitudinal investigation, a significant decline was observed in the levels of phosphate and calcium in breast milk over time, with concentrations of phosphate in breast milk at 3, 6, and 26 weeks of lactation of 14.7, 12.7, and 10.7 mg/100 ml, respectively.^26^ Corresponding values for calcium concentrations in breast milk during those time frames were 25.9, 27.7, and 24.8 mg/100 ml, respectively.^26^ Another possible explanation is that the prophylactic effects of breast milk against dental caries (through the transfer of maternal protective elements such as immunoglobulins, lactoferrin, and casein^27^ from mother to infant) decline due to the gradual depletion of these elements after prolonged lactation.^9^ Alternatively, some unknown factors related to longer breastfeeding may have confounded the observed association. Mothers who breastfeed longer may be more likely to sleep with their child and breastfeed freely during the night.^17^ A cross-sectional study of Brazilian preschool children showed that the prevalence of early childhood caries was higher in children who were breastfed at night after age 12 months than in children who stopped breastfeeding before age 12 months.^18^

Our study has several strengths. The study subjects were similar in age and geographic background, which likely reduced potential confounding produced by unmeasured factors related to age and geographic background. Data on dental caries were obtained by means of oral examinations by dentists, and we were able to control for a variety of potential confounding factors.

This study also has some limitations that should be considered. Of the 8269 eligible subjects in Fukuoka City, only 2057 (24.9%) were included in this analysis, so selection bias may be a factor. We were unable to assess differences between participants and nonparticipants because, except for age, no information on the personal characteristics of nonparticipants was available. Thus, our subjects cannot be considered representative of Japanese children in the general population, and the present findings should not be generalized. In fact, the educational levels of parents in the present study were higher than those of the general population. According to the 2000 population census of Japan, the proportions of men aged 35 to 39 years in Fukuoka City with less than 13 years, 13 to 14 years, 15 years or more, and unknown years of education were 39.6%, 8.0%, 43.3%, and 9.1%, respectively.^28^ The corresponding figures among fathers in the present study were 27.5%, 14.9%, 57.6%, and 0.0%, respectively. The proportions of women 30 to 34 years of age in Fukuoka City with less than 13 years, 13 to 14 years, 15 years or more, and unknown years of education were 41.3%, 34.4%, 16.1%, and 8.3%, respectively.^28^ The corresponding figures among mothers in the present study were 28.3%, 40.1%, 31.7%, and 0.0%, respectively. In addition, the prevalence of dental caries in the study population (20.7%) was lower than that in a sample of 3-year-old Japanese children assessed in a 2005 survey of dental diseases (24.4%).^29^

The data on dental caries used in the present study were gathered during routine examinations by a number of dentists at public health centers. The dentists were given detailed criteria for performing the examination but were not specifically trained so as to ensure standardization of their examinations. The unstandardized nature of the examinations could lead to nondifferential misclassification of caries and thus bias the results toward the null, that is, toward a lack of association between breastfeeding and early childhood caries. Moreover, because parents or guardians of the children transcribed the data gathered at the dental examinations from their Maternal and Child Health Handbook to our self-administered questionnaire, we cannot exclude the possibility that transcription errors occurred. Nevertheless, misclassification of outcome is unlikely to differ across categories of breastfeeding status. If this had occurred, the consequence would have been an underestimation of values in our results. Breastfeeding duration was assessed 3 years after the birth of the children, and this delay could have led to recall bias. Most questionnaires, however, were completed by the child’s mother, and it is unlikely that a mother would not remember the breastfeeding history of her child. In a validation study, breastfeeding duration reported by the mother has been shown to be accurate for 20 years or even longer after the birth of a child.^30^ Even if errors in measurement of breastfeeding duration occurred, misclassification is unlikely to differ across exposure categories. If this had occurred, the results would be biased toward the null.

Although we have adjusted our analyses for numerous potential confounders, such as toothbrushing frequency, use of fluoride, and parental educational levels, residual confounding effects cannot be ruled out. Additionally, it is possible that our results remain confounded by other potentially important factors such as frequency of bedtime breastfeeding, age at introduction of foods and fluids other than breast milk, and dietary intake (eg, sugar intake). Our study design was cross-sectional, so the results of this analysis should not be interpreted as providing evidence of a cause-effect relationship between breastfeeding and dental caries.

In conclusion, we found a J-shaped association between breastfeeding duration and the prevalence of dental caries among young Japanese children. Our findings indicate that breastfeeding for 18 months or longer is associated with a higher prevalence of dental caries. From the perspective of avoiding any harmful impacts of breastfeeding on infant dental health, the ideal duration of breastfeeding might be shorter than 18 months, given that a nonsignificant inverse association was observed between breastfeeding for 6 to 17 months and the prevalence of dental caries. Further, prospective, studies with clearly defined breastfeeding variables (eg, exclusive breastfeeding duration and breastfeeding frequency) that also account for confounding factors need to be undertaken before a definitive conclusion can be reached.
